# COVID-19 and Pregnancy: Citation Network Analysis and Evidence Synthesis

**DOI:** 10.2196/29189

**Published:** 2022-03-03

**Authors:** Rebeca Ruiz-Roman, Clara Martinez-Perez, Inés Gil Prados, Ignacio Cristóbal, Miguel Ángel Sánchez-Tena

**Affiliations:** 1 Department of Gynecology and Obstetrics Hospital Clínico San Carlos Madrid Spain; 2 Instituto Superior de Educação e Ciências Lisboa Portugal; 3 Faculty of Medicine Universidad Francisco de Vitoria Madrid Spain; 4 Department of Optometry and Vision Faculty of Optics and Optometry Universidad Complutense de Madrid Madrid Spain

**Keywords:** pandemic, COVID-19, SARS-CoV-2, pregnancy, perinatal, citation, bibliometric, network analysis, women, maternal health, fetal health, research, literature, transmission, delivery, impact

## Abstract

**Background:**

COVID-19 spread quickly around the world shortly after the first outbreaks of the new coronavirus disease at the end of December 2019, affecting all populations, including pregnant women.

**Objective:**

The aim of this study was to analyze the relationship between different publications on COVID-19 in pregnancy and their authors through citation networks, as well as to identify the research areas and to determine the publication that has been the most highly cited.

**Methods:**

The search for publications was carried out through the Web of Science database using terms such as “pregnancy,” “SARS-CoV-2,” “pregnant,” and “COVID-19” for the period between January and December 2020. Citation Network Explorer software was used for publication analysis and VOSviewer software was used to construct the figures. This approach enabled an in-depth network analysis to visualize the connections between the related elements and explain their network structure.

**Results:**

A total of 1330 publications and 5531 citation networks were identified in the search, with July being the month with the largest number of publications, and the United States, China, and England as the countries with the greatest number of publications. The most cited publication was “Clinical characteristics and intrauterine vertical transmission potential of COVID-19 infection in nine pregnant women: a retrospective review of medical records” by Chen and colleagues, which was published in March 2020. Six groups identified as being close in the citation network reflect multidisciplinary research, including clinical characteristics and outcomes in pregnancy, vertical transmission, delivery mode, and psychological impacts of the pandemic on pregnant women.

**Conclusions:**

Thousands of articles on COVID-19 have been published in several journals since the disease first emerged. Identifying relevant publications and obtaining a global view of the main papers published on COVID-19 and pregnancy can lead to a better understanding of the topic. With the accumulation of scientific knowledge, we now know that the clinical features of COVID-19 during pregnancy are generally similar to those of infected nonpregnant women. There is a small increase in frequency of preterm birth and cesarean birth, related to severe maternal illness. Vaccination for all pregnant women is recommended. Several agents are being evaluated for the treatment of COVID-19, but with minimal or no information on safety in pregnancy. These results could form the basis for further research. Future bibliometric and scientometric studies on COVID-19 should provide updated information to analyze other relevant indicators in this field.

## Introduction

In late December 2019, a cluster of novel human pneumonia cases in Wuhan City, China, were reported. Shortly after, on January 12, 2020, the World Health Organization temporarily termed the new virus “2019 novel coronavirus” (2019-nCoV) and then officially named this infectious disease COVID-19 on February 12, 2020, becoming the fifth pandemic after the 1918 flu pandemic, affecting people all over the world [1]. As of December 12, 2021, nearly 269 million confirmed cases and nearly 5.3 million deaths were reported globally. Recent reports of different variants of SARS-CoV-2 have raised concern and interest in the impact of viral changes [2].

Coronaviruses (Coronaviridae) are a family of viruses that cause infections in humans and animals. That is, coronavirus infections are considered to be zoonotic diseases that can be transmitted from animals to humans through direct contact with infected animals or their secretions. SARS-CoV-2, which causes COVID-19, exhibits human-to-human transmission by multiple means, namely by droplets, aerosols, and fomites [3]. Knowledge on COVID-19 in pregnant women has evolved tremendously; earlier reports from China considered the possibility of vertical transmission, but subsequent reports depicted a theoretical risk, and increased prevalence of preterm deliveries was also noticed [4].

The most frequent clinical characteristics of COVID-19 consist of fever, cough, fatigue or myalgia, sputum production, and headache [5]. Although viral pneumonia is an important cause of morbidity and mortality among pregnant women, the most common manifestations of COVID-19 during pregnancy are similar to those of infected nonpregnant women. However, infected mothers may be at increased risk for more severe respiratory complications [6,7].

Besides affecting the respiratory tract, COVID-19 has a remarkable impact on the mental health of pregnant women, since they are already at increased risk of developing mental health problems such as depression, anxiety, and posttraumatic stress symptoms [8]. This should be taken into account since it is proposed that the psychological stress of the COVID-19 pandemic during pregnancy can increase the risk of neurodevelopmental disorders in offspring [9]. Hence, it is important to proactively develop appropriate strategies to alleviate stress by screening, identifying, and managing perinatal mental health disorders during the pandemic.

Regarding the treatment of COVID-19, most guidelines include oxygen therapy, antiviral therapy, and supportive treatment. To date, dexamethasone is the only proven and recommended experimental treatment for pregnant patients with COVID-19 who are mechanically ventilated or who require supplemental oxygen [10]. Several other drugs are being used in research studies (eg, antiviral drugs, monoclonal antibodies, immunomodulators), but very few trials include pregnant people.

Citation networks enable searching for scientific literature on a specific topic. That is, by means of a publication, other relevant publications can be sought to demonstrate, qualitatively and quantitatively, the relationships between articles and authors through the creation of groups [11]. This approach further enables quantifying the most cited publications in each group and studying the development of a research area or focusing the bibliographic search on a specific topic [12,13].

Great efforts in knowledge production about the COVID-19 pandemic caused by the SARS-CoV-2 virus have been made from the beginning of the outbreak. In early 2020, studies about the scientific literature on COVID-19 and bibliometric analyses were published to summarize the research hotspots and offer a review of the topic to provide a reference for researchers. From inception (ie, the beginning of the pandemic) to March 1, 2020, the first authors of these publications were from 20 different countries and the papers were published in 80 different journals [14]. A bibliometric analysis of publications in five high-impact journals indexed to the Web of Science Core Collection’s Science Citation Index Expanded (SCI-EXPANDED) database was also published [15]. By June 2020, China, the United States, and the United Kingdom were the most represented countries, and *The Lancet* was the journal with the highest number of contributions on the topic [15,16]. In Italy, a systematic review and bibliometric analysis of the scientific literature on the early phase of COVID-19 was conducted, but with limited international impact [17]. Furthermore, citation networks were used to investigate the strategic themes, thematic evolution structure, and trends of publications during the first 8 months of the COVID-19 pandemic in the Web of Science database in 2020, providing new perspectives of the field [18]. This tool has also been used in the pediatrics literature to identify publication trends and topic dissemination, showing the relevance of the publishing authors, institutions, and countries [19]. Most recently, the interdisciplinary status of coronavirus-related fields was investigated via the COVID-19 Open Research Dataset (CORD-19). To this end, bibliometric indicators of interdisciplinarity were calculated and a cooccurrence analysis method was applied [20]. Interdisciplinary research can provide an effective solution to complex issues in the related field of coronavirus research.

Taking into account the significant increase in the number of publications on COVID-19 and pregnancy, the aim of this study was to analyze the relationship between different publications and their authors through citation networks, as well as to identify the research areas and determine which publication has been the most highly cited. For this purpose, the analysis was carried out using Citation Network Explorer (CitNetExplorer) software.

## Methods

### Database

The following search terms were used to search the publications in the Web of Science database: “pregnancy,” “SARS-CoV-2,” “pregnant,” and “COVID-19.” These terms were selected according to the main objective of this study because they are the most common terms in all related research fields.

Given that the search results contained articles in common, the Boolean operators NOT and AND, along with the “*” character, were used to find the singular and plural forms of the words. In this way, the terms used were ([COVID-19 OR SARS-CoV-2] AND [pregnancy OR pregnant]). Likewise, the search field was selected by topic, thereby limiting the search by abstract, title, and keywords. The selected time interval was from January 2020 to December 2020.

In turn, Web of Science also makes it possible to add references to your library while performing bibliographic searches directly in external databases or library catalogs. With regard to the citation index, the Social Sciences Citation Index, SCI-EXPANDED, and Emerging Sources Citation Index were used. However, because of the different citation methods used by various authors and organizations, CiteSpace software was used to standardize the data. The publications were searched and downloaded on November 23, 2020.

### Network Analysis

The publications were analyzed using CitNetExplorer software. This software allows for the analysis and visualization of the citation networks of scientific publications, and further allows for citation networks to be downloaded directly from Web of Science. It is also possible to manage citation networks including thousands of interrelated publications and citations. As such, researchers can use a citation network consisting of thousands of publications as the starting point, before going on to perform a deeper analysis of the most relevant publications to generate a small subnetwork containing ~100 publications on the same topic. The Citation Score attribute was used to perform quantitative analysis on the most cited publications within a specific time frame. In this way, both internal connections within the Web of Science database and external connections were quantified, meaning that other databases were also considered [13].

CitNetExplorer offers several techniques for analyzing the different citation networks. The *clustering function* is achieved using the formula developed by Van Eck and Waltman [13] in 2021. The clustering function was used to assign a group to each publication. As a result, the most interrelated publications tended to be within the same group based on the citation networks [13].

Finally, the main publications were analyzed using the *identifying core publications* function. This function is based on identifying the publications that are considered as the core of a citation network (ie, those with a minimum number of connections with other core publications), making it possible to eliminate publications that are considered unimportant. The number of connections is established by the researchers, so that a higher value of this parameter indicates a lower number of core publications [13]. Thus, in this study, the publications with four or more citations within the citation network were taken into account. The *drilling down* function was used since it allows for a deeper analysis of each group at different levels. VOSviewer software (version 1.6.9) was used for generating figures [21,22].

With this approach, it was possible to carry out an in-depth network analysis to visualize the connections between the related elements and explain their network structure. We considered three main subnetworks: a country coauthorship network, cited references cocitation network, and author keyword cooccurrence network.

The coauthorship analysis allowed for the identification of collaboration networks between countries in this field of research. The nodes correspond to the countries participating in this field of research and the links between the elements imply cooperative relationships. The size of the node increases in parallel with an increase in the number of articles published by an individual country. The number of links shows the number of times that a given country shared coauthorship with others. Therefore, the strength of the link increases as the number of coauthors increases.

In the cocitation network, nodes represent scientific references and the size of the nodes represents the number of times a reference is cited. The correlation of the articles according to the cocitation links was represented according to the distance between two references. Self-citations were not considered for analysis.

In the author keyword cooccurrence network, nodes represent the most frequently cited author keywords, and the size of an individual node represents how many times that keyword is cited. The strength of the link between two nodes indicated the number of articles in which two keywords appeared together [21,22].

Each group is determined by a resolution value, which ranges from 1.0 to 0.50 [21,22].

### Scientometric Analysis

CiteSpace (5.6.R2) software was used to perform the scientometric analysis. This software, developed by Chen Chaomei, is Java-based and is comprised of five basic theoretical aspects: Kuhn’s model of scientific revolutions, Price’s scientific frontier theory, the organization of ideas, the best information-foraging theory of scientific communication, and the theory of discrete and reorganized knowledge units [23,24]. In the scientometric analysis process, there are also some parameter indicators to carry out a specific assessment. The H-index is a mixed quantitative index, suggested by George Hirsch from the University of California, which is used to evaluate the quantity and level of the academic output of researchers and institutions. The H-index indicates that *h* out of *N* published articles in a journal have been cited at least *h* times [25]. The degree indicates the number of connections that exist among the authors (organizations, countries) in the cooccurrence knowledge graph. A higher degree value indicates a greater level of communication and collaboration between the authors (organizations, countries). The centrality value measures the importance of the nodes within the research cooperation network. Intermediary centrality is a measure of the number of times a node acts as a waypoint along the shortest path between two other nodes, according to the geodetic distance. The half-life is a parameter that represents the continuity of institutional research from a time perspective [23], which is defined as the number of years a publication receives half of its citations since it was published. A low citation half-life suggests citation activity that peaks and declines rapidly. A high half-life suggests citation activity that peaks and then declines more slowly.

## Results

### Publication Trend

Since the first articles on COVID-19 were published at the beginning of 2020, the period of time selected for this analysis was from January 2020 to December 2020. A total of 1330 publications and 5531 citation networks were found in the search in all fields on Web of Science. Among all 1330 publications, 721 (54.21%) were articles, 268 (20.15%) were letters to the editor, 185 (13.91%) were reviews, 121 (9.10%) were letters, 17 (1.28%) were meeting abstracts, 9 (0.68%) were news items, 5 (0.38%) were corrections, and 4 (0.30%) were proceedings papers.

**Figure 1:**
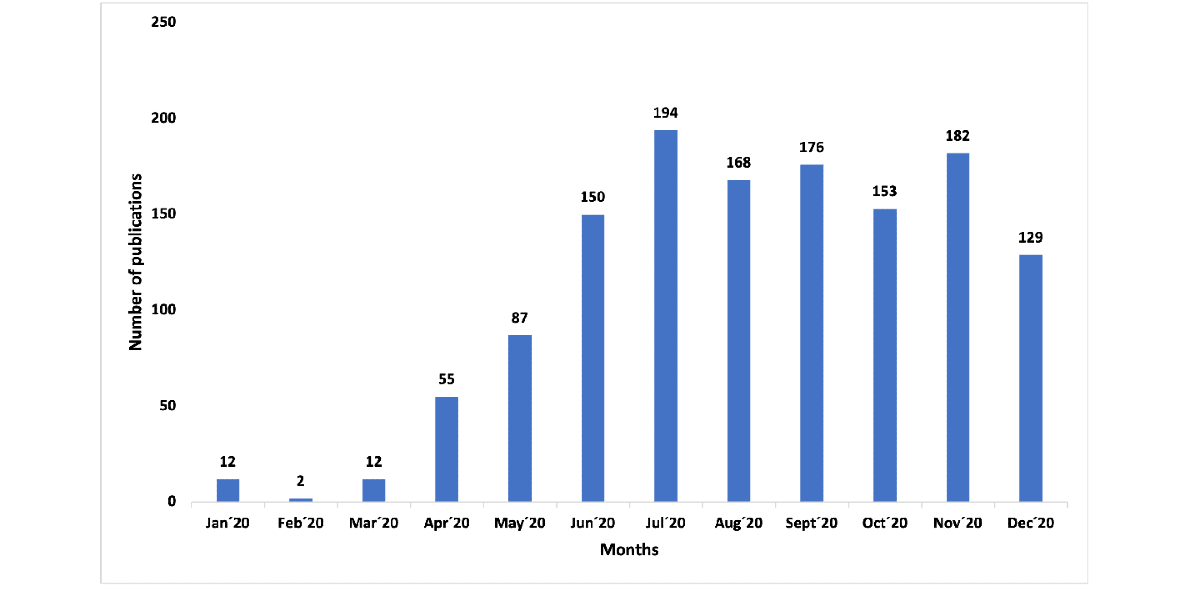
Number of publications per month.

The number of publications on COVID-19 has increased since May 2020 (January to April 2020: 9.26% of the total publications; May to December 2020: 90.74% of the total publications). July was the month with the largest number of publications, with 194 publications and 27 citation networks (Figure 1). A detailed description of the publications is provided in Multimedia Appendix 1.

### Most Cited Publications

The most cited article was that of Chen et al [26], published in March 2020, with a citation index of 279. The aim of their study was to evaluate the clinical characteristics of COVID-19 in pregnancy and the potential for the vertical intrauterine transmission of COVID-19 infection. To this end, clinical records, laboratory results, and chest computed tomography (CT) scans of nine pregnant women with COVID pneumonia who were admitted to Zhongnan Hospital of Wuhan University, Wuhan, China, from January 20 to 31, 2020, were reviewed retrospectively. Evidence of vertical intrauterine transmission was evaluated by testing for SARS-CoV-2 in amniotic fluid, cord blood, and neonatal pharyngeal smear samples. Breastmilk samples were also collected and analyzed from patients after the first breastfeeding. The results showed that all nine patients had a cesarean section in their third trimester. Seven patients presented with fever. Other symptoms were also observed, including cough (in four of the nine patients), myalgia (in three patients), sore throat (in two patients), and malaise (in two patients). Fetal distress was controlled in two cases. Five of the nine patients had lymphopenia and three patients had increased aminotransferase concentrations. However, none of the patients developed severe pneumonia from COVID-19. Nine live births were recorded and no neonatal asphyxia was observed in newborns. All nine live births had an Apgar score of 8 to 9 at 1 minute and an Apgar score of 9 to 10 at 5 minutes. The clinical characteristics of COVID-19 pneumonia in pregnant women were similar to those reported for pregnant patients who did not develop COVID-19 pneumonia. In conclusion, the findings suggest that there is no evidence of intrauterine infection caused by vertical transmission in women with COVID-19 in late pregnancy.

Among the 20 most cited articles (Table 1), 18 address the clinical manifestations, and obstetric and neonatal outcomes of pregnant patients with COVID-19. They also refer to the vertical transmission of COVID-19 in late pregnancy, including vaginal delivery. The remaining 2 articles deal with how to evaluate the management and safety of epidural or general anesthesia for cesarean delivery in women with COVID-19 and their newborns, and evaluation of standardized procedures for the protection of medical staff.

**Table 1:** Top 20 most cited articles about COVID-19 and pregnancy (January to December 2020).

Author	Title	Journal	Citation index
Chen et al [26]	Clinical characteristics and intrauterine vertical transmission potential of COVID-19 infection in nine pregnant women: a retrospective review of medical records	The Lancet	436
Rasmussen et al [27]	Coronavirus Disease 2019 (COVID-19) and pregnancy: what obstetricians need to know	American Journal of Obstetrics and Gynecology	159
Schwartz et al [28]	Potential maternal and infant outcomes from (Wuhan) coronavirus 2019-nCoV infecting pregnant women: lessons from SARS, MERS, and other human coronavirus infections	Viruses	154
Breslin et al [29]	Coronavirus disease 2019 infection among asymptomatic and symptomatic pregnant women: two weeks of confirmed presentations to an affiliated pair of New York City hospitals	American Journal of Obstetrics and Gynecology	144
Yu et al [30]	Clinical features and obstetric and neonatal outcomes of pregnant patients with COVID-19 in Wuhan, China: a retrospective, single-centre, descriptive study	Lancet Infectious Diseases	130
Schwartz [31]	An analysis of 38 pregnant women with COVID-19, their newborn infants, and maternal-fetal transmission of SARS-CoV-2: maternal coronavirus infections and pregnancy outcomes	Archives of Pathology & Laboratory Medicine	123
Dashraath et al [32]	Coronavirus disease 2019 (COVID-19) pandemic and pregnancy	American Journal of Obstetrics and Gynecology	118
Zaigham et al [33]	Maternal and perinatal outcomes with COVID-19: A systematic review of 108 pregnancies	Acta Obstetricia et Gynecologica Scandinavica	107
Di Mascio et al [34]	Outcome of coronavirus spectrum infections (SARS, MERS, COVID-19) during pregnancy: a systematic review and meta-analysis	American Journal of Obstetrics and Gynecology	92
Mullins et al [35]	Coronavirus in pregnancy and delivery: rapid review	Ultrasound in Obstetrics & Gynecology	87
Yan et al [36]	Coronavirus disease 2019 in pregnant women: a report based on 116 cases	American Journal Obstetrics and Gynecology	80
Alzamora et al [37]	Severe COVID-19 during pregnancy and possible vertical transmission	American Journal of Perinatology	78
Wang et al [38]	A case report of neonatal 2019 coronavirus disease in China	Clinical Infectious Diseases	75
Qiao et al [39]	What are the risks of COVID-19 infection in pregnant women?	The Lancet	74
Wang et al [40]	A case of 2019 novel coronavirus in a pregnant woman with preterm delivery	Clinical Infectious Diseases	74
Knight et al [41]	Characteristics and outcomes of pregnant women admitted to hospital with confirmed SARS-CoV-2 infection in UK: national population based cohort study	BMJ	74
Hantoushzadeh et al [42]	Maternal death due to COVID-19	American Journal of Obstetrics and Gynecology	74
Baud et al [43]	Second-trimester miscarriage in a pregnant woman with SARS-CoV-2 infection	JAMA	65
Liang et al [44]	Novel corona virus disease (COVID-19) in pregnancy: what clinical recommendations to follow?	Acta Obstetricia et Gynecologica Scandinavica	61
Ellington et al [45]	Characteristics of women of reproductive age with laboratory-confirmed SARS-CoV-2 infection by pregnancy status - United States, January 22-June 7, 2020	Morbidity and Mortality Weekly Report	61

### Clustering Function

**Figure 2:**
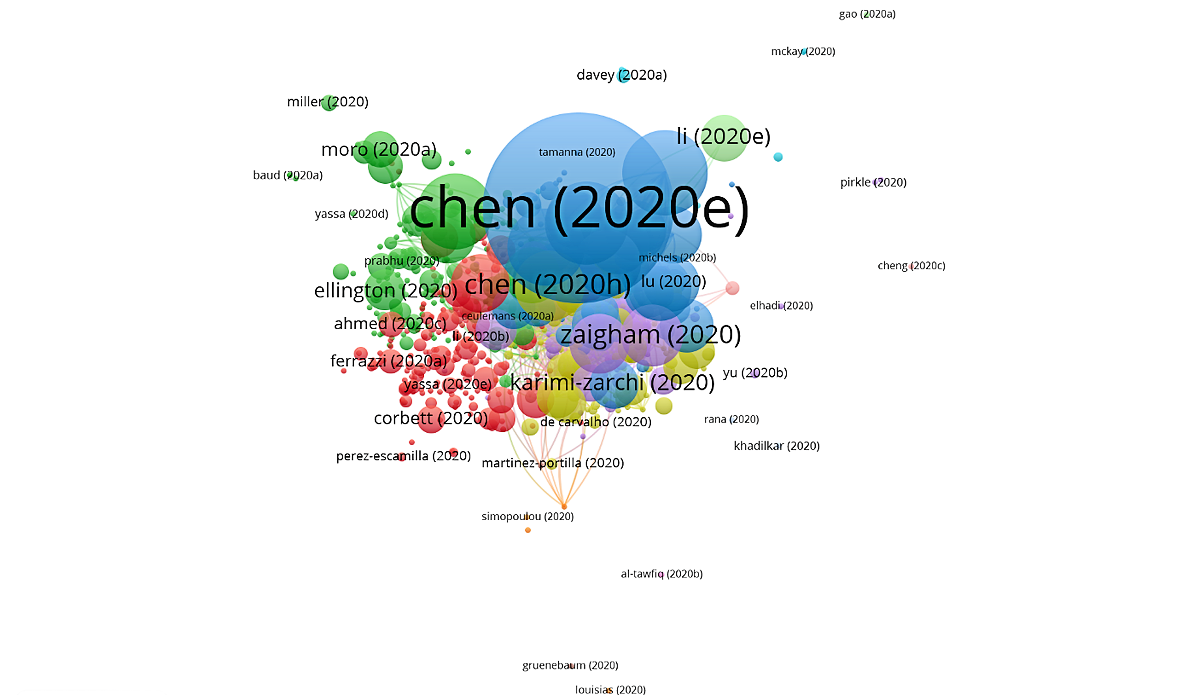
Clustering function in the citation network on COVID-19 and pregnancy.

Using the clustering function, each publication in the citation network is assigned to a group, which means that publications that are close in the citation network must belong to the same group. Consequently, each of these groups consists of publications that are strongly connected through their citations. In this way, it could be interpreted that every group represents a different topic in the scientific literature. To differentiate among groups, each group was assigned a specific color. Additionally, the links between groups have been marked using colored lines. The clustering function identified 6 groups, 4 of which contained a significant number of articles; however, the remaining groups only accounted for 5.72% of the total number of citations (Figure 2). The citation networks show the publications with the highest weight and the group to which they belong. The size of the circle increases with the increase in the number of citations. The color of an article represents its group and the lines that connect the elements represent links. Thus, the articles of the same group will have the same color. Table 2 shows the information of the citation networks regarding the 4 main groups, listed from the largest to the smallest according to their size.

**Figure 3:**
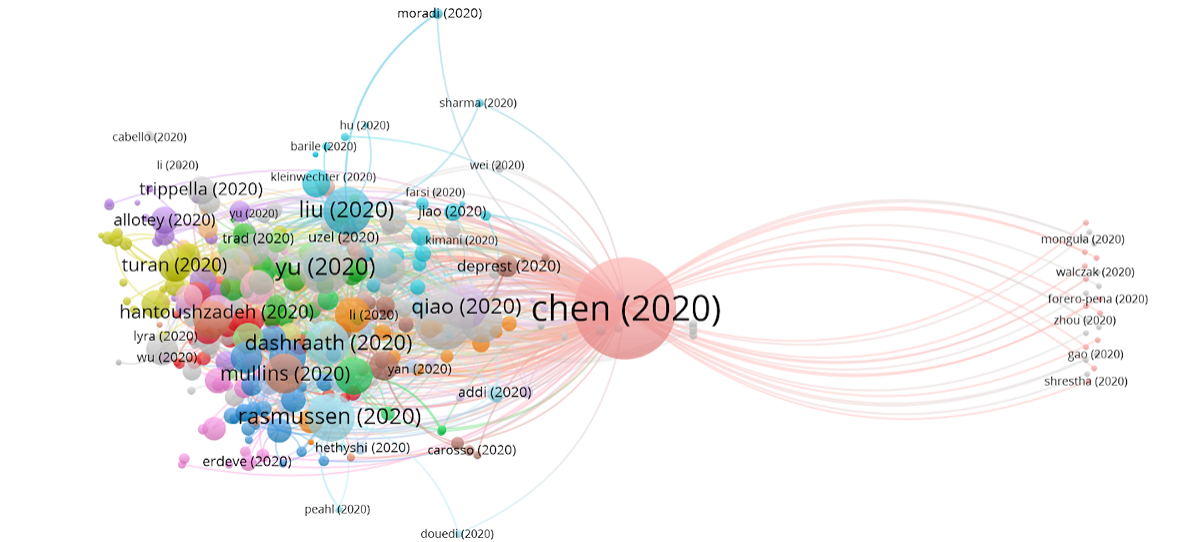
Citation network for Group 1.

In group 1, 757 articles and 4407 citations were identified throughout the network. The most cited publication was that of Chen et al [26], published in March 2020 in *The Lancet*, which also ranked first among the 20 most cited publications. The publications of this group are focused on describing the clinical manifestations, and the obstetric and neonatal outcomes of pregnant patients with COVID-19. They also address the vertical transmission topic of COVID-19 in late pregnancy, including vaginal delivery (Figure 3) and treatment with chloroquine and hydroxychloroquine in pregnant women.

**Figure 4:**
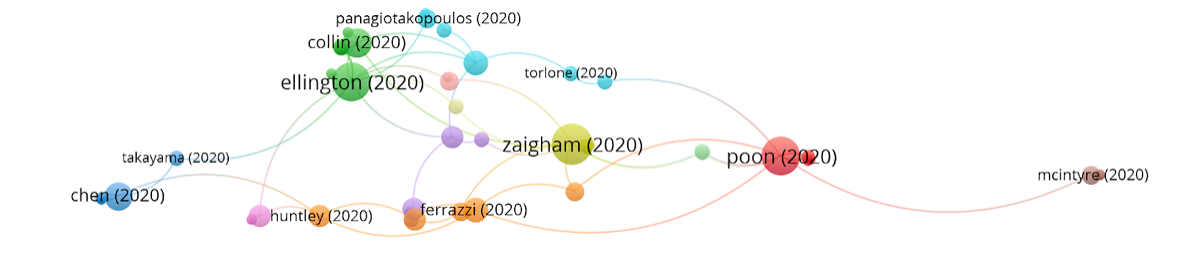
Citation network for Group 2.

In group 2, 106 publications and 192 citations were identified throughout the network. The most cited publication was that of Zaigham et al [33], published in April 2020 in *Acta Obstetricia et Gynecologica Scandinavica*. The aim of this study was to summarize the clinical manifestations, and maternal and perinatal outcomes of COVID-19 during pregnancy. To accomplish this, the databases were searched using multiple terms and combinations: COVID-19, pregnancy, maternal mortality, maternal morbidity, complications, clinical manifestations, neonatal morbidity, intrauterine fetal death, neonatal mortality, and SARS-CoV-2. The results showed that most reports describe pregnant women in the third trimester with fever (68%) and cough (34%). Lymphocytopenia (59%) with elevated C-reactive protein (70%) was also observed, and 91% of the women delivered by cesarean section. One neonatal death and one intrauterine death were also found. Therefore, it is necessary to monitor pregnant women with COVID-19 and try to prevent neonatal infection. This group’s publications also address how to evaluate the management and safety of epidural or general anesthesia for cesarean delivery in the context of COVID-19, and how to evaluate standardized procedures for the protection of medical staff. Besides avoiding nosocomial infections, recommendations for testing for gestational diabetes mellitus and the presence of sickle cell disease in patients with COVID-19 are provided (Figure 4).

**Table 2:** Information on the citation network of the 4 main groups.

Main cluster	Number of publications	Number of citation links	Number of citations, median (range)	Number of publications with ≥4 citations	Number of publications in 100 most cited publications
Group 1	757	4407	0 (0-436)	506	91
Group 2	106	192	0 (0-107)	23	4
Group 3	32	56	0 (0-51)	9	4
Group 4	29	30	0 (0-25)	4	1

**Figure 5:**
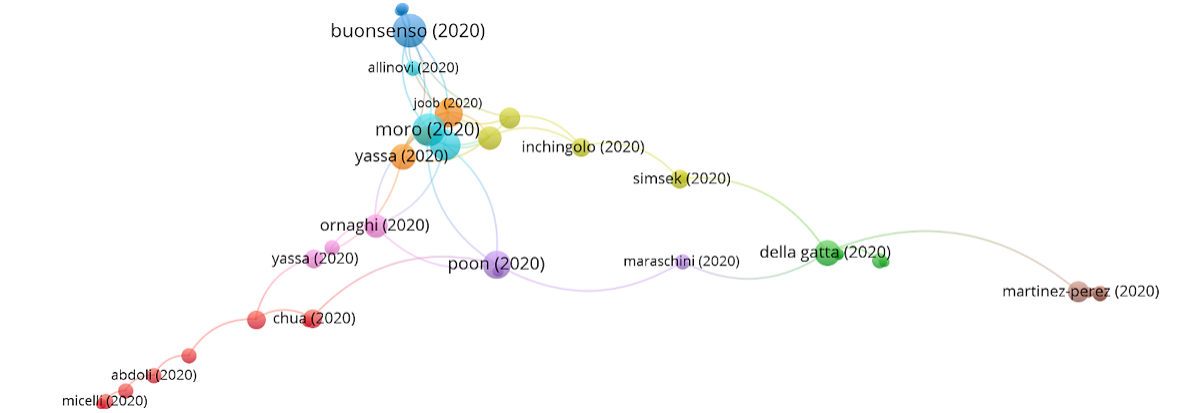
Citation network for Group 3.

In group 3, 32 publications and 56 citations were identified throughout the network. The most cited publication was Della et al [46], published in July 2020 in *American Journal of Obstetrics and Gynecology*. The aim of this study was to perform a systematic review of reported clinical outcomes for pregnant patients with COVID-19. All studies of pregnant women who received a diagnosis of COVID-19 through a nucleic acid test, with reported data on pregnancy and delivery cases, were included. Among 51 pregnant women, 3 pregnancies were in progress. Of the remaining 48 pregnant women, 46 delivered by cesarean section and 2 delivered vaginally. In turn, 1 stillbirth and 1 neonatal death were reported in this study. In conclusion, although vertical transmission of COVID-19 infection has been excluded to date, and the outcome for mothers and newborns has been generally good, the high rate of premature cesarean deliveries is a cause for concern. Cesarean delivery was typically an elective surgical intervention, and it is reasonable to question whether cesarean delivery for pregnant patients with COVID-19 was justified. COVID-19 and respiratory failure association in late pregnancy certainly creates a complex clinical scenario. The publications of this group address the significant increase in the rate of cesarean deliveries (>90%) and whether the mode of delivery is associated with maternal complications or neonatal transmission. These studies also emphasize the importance of imaging modalities in the treatment of patients suspected of having COVID-19, highlighting pulmonary ultrasound. This can be a valid alternative to chest CT, especially for pregnant women, since it presents certain advantages: the ultrasound can be performed directly at the bedside by only one professional, which reduces the risk of spreading the disease. In addition, it is a test without radiation, which makes it safer and easier when monitoring patients who require a series of tests (Figure 5).

**Figure 6:**
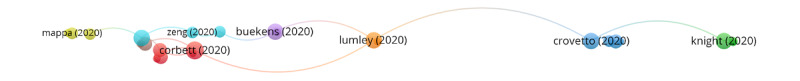
Citation network for Group 4.

In group 4, 29 publications and 30 citations were identified throughout the network. The most cited publication was that of Corbett et al [47], published in August 2020 in *American Journal of Obstetrics and Gynecology*. The aim of this study was to examine the influence of COVID-19 on the prevalence of symptoms of depression and anxiety, as well as the corresponding risk factors among pregnant women in China. The results showed that pregnant women after the declaration of a pandemic had a higher rate of depressive symptoms than women evaluated previous to the appearance of COVID-19. These women are also at higher risk of thoughts of self-harm. It should be noted that women who were underweight before pregnancy, primiparous, under 35 years of age, and employed full time had a greater risk of developing depressive and anxiety symptoms during the disease. In conclusion, major life-threatening public health events such as COVID-19 increase mental health problems during pregnancy. Therefore, communication and the provision of psychological first aid can be useful to prevent negative results. Publications from this group assess the psychological impact of the COVID-19 pandemic on pregnant women (Figure 6).

**Figure 7:**
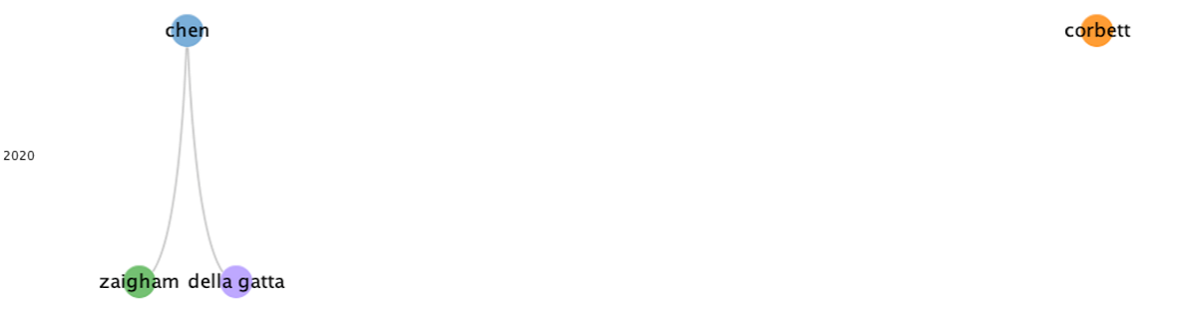
Relation among the 4 main groups in the citation network.

When analyzing the relationship between groups, a connection was found between groups 1, 2, and 3. Therefore, these groups analyze topics that are clearly related to each other (Figure 7).

### Subclusters in Group 1

Six subclusters were found in group 1, three of which have a significant number of publications (Table 3). The rest of the groups are relatively small, with fewer than 82 publications and 138 citation networks.

**Table 3:** Main citation network groups from the subclusters of Group 1.

Characteristic	Subcluster 1	Subcluster 2	Subcluster 3
Number of publications	430	241	35
Number of citation links	2002	979	36
Most cited publication	Chen et al [26]	Ferrazi et al [48]	Li et al [49]
Main keywords	COVID-19, vertical transmission, infection	neonatal, virus, labor	SARS-CoV-2, pregnancy, prenatal care
Topic of discussion	Assess clinical characteristics and outcomes in pregnancy and the potential for vertical transmission of COVID-19 infection	Report vaginal delivery or cesarean section and immediate neonatal outcome in women infected with COVID-19	Impact of SARS-CoV-2 on male reproduction and pregnancy outcomes
Conclusion	COVID-19 infection during pregnancy is not associated with an increased risk of miscarriage or premature spontaneous birth. There is no evidence of vertical transmission of COVID-19 infection when it occurs during the third trimester of pregnancy.	Although postpartum infection cannot be excluded with 100% certainty, these findings suggest that vaginal delivery is associated with a low risk of intrapartum transmission of SARS-Cov-2 to the newborn. In addition, SARS-CoV-2 has been found in breastmilk.	Male gonads may be potentially vulnerable to SARS-CoV-2 infection, so caution is advised for pregnant women and couples planning a natural pregnancy or assisted reproduction.

### Core Publications

**Figure 8:**
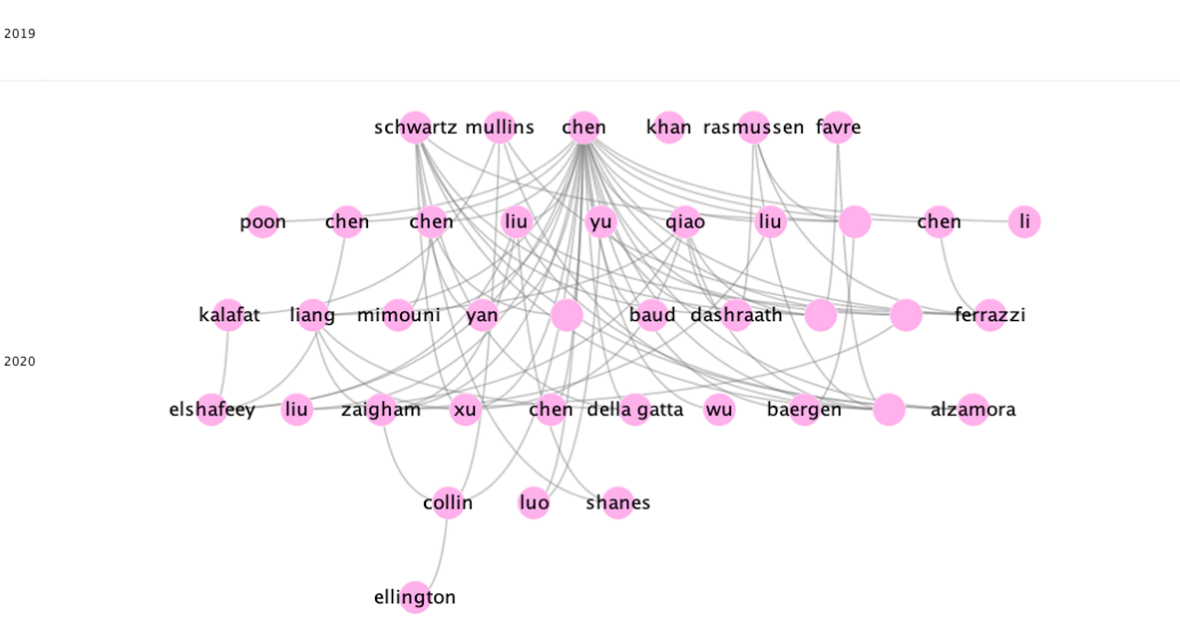
Core publications in the citation network of COVID-19 and pregnancy.

We found 610 publications with 4 or more citations and the citation network included 4726 citations, representing 45.86% of the 1330 total publications retrieved. This indicates the diversity of the research topic, showing that many research topics are analyzed. However, there is a clear focus in scientific investigation carried out in this field. In this analysis, the main topic was to describe the clinical manifestations, and the obstetric and neonatal outcomes of pregnant patients with COVID-19, along with the vertical transmission of COVID-19 in the late stage of pregnancy and at the time of delivery (Figure 8).

## Discussion

### Principal Findings

The main databases such as Web of Science or Scopus allow for the creation of citation networks. On the one hand, Scopus includes a greater variety of publications compared to PubMed and Web of Science. On the other hand, the citation analysis presented by Web of Science offers better graphics and is more detailed than that performed by Scopus. This is likely because Web of Science was developed for the purpose of analyzing citations. PubMed focuses on the clinical and biomedical literature, whereas Web of Science is interdisciplinary, and includes journals of the highest quality in each subject area [13]. However, the usefulness of some of these databases is limited to conducting a systematic review of the literature, since they fail to offer an overview of the connections between citations in a group of publications. This is the main reason that we used CitNetExplorer and CiteSpace software in this study, since these tools enable visualizing, analyzing, and exploring citation networks in scientific publications [13]. In this way, CitNetExplorer obtains a more detailed analysis through the creation of dating networks (ie, networks based on the date of publication) compared to other databases.

Web of Science is one of the most extensive databases, beginning its search framework in 1900. However, as a limitation of this study, Web of Science only accepts international journals and its selection process is exhaustive, which means that some publications that are not in Web of Science could have been excluded from this analysis.

The main goal of this study was to analyze the existing literature on COVID-19 and pregnancy. To identify relevant publications, the Web of Science database was used, and then connections between the fields of study and different research groups were analyzed. To obtain the results, the *clustering* function was used. This function allows for publications to be grouped according to the relationships that exist among citations. The *drilling down* function was also used to perform a more in-depth analysis of the existing bibliography for each group. The *core publications* function shows the main publications (ie, those with a minimum number of citations). Therefore, these functions make it possible for a complete analysis of the research in the field of interest.

Most of the knowledge about how a coronavirus infection might affect pregnant patients comes from the previous severe acute respiratory syndrome (SARS) and Middle Eastern respiratory syndrome (MERS) epidemics. These experiences showed that coronavirus infections could increase the risk of life-threatening maternal illness, intrauterine growth restriction, preterm delivery, miscarriage, and perinatal death [28]. Since the SARS-CoV-2 pandemic was declared, numerous case reports and reviews have been published regarding this new pathogen and its role in the course of pregnancy. The month with the largest number of publications on “COVID-19 and pregnancy” was July, which was only 1 month later in comparison to articles issued on “COVID-19” [50]. The article with the highest citation index was published by Chen et al [26] in March 2020. The countries with the largest number of research articles published on the topic are the United States, China, and England. It is logical that most articles written at the beginning of the pandemic were by Chinese researchers. However, this has been a cause of major concern among frontline health workers and politicians due to the language barrier. Currently, the English language and high investment in research likely justifies why the United States and England are among the top 3 countries in terms of the number of publications. Another factor linked to this finding is the possibility to make connections between different research groups within the scientific community [51].

Clinical manifestations and obstetric and perinatal outcomes have been the main concern in this field, which have been addressed in 18 of the 20 most cited articles on COVID-19 and pregnancy. Most of these studies state that there are no differences in the symptomatology compared with that of nonpregnant patients. The article with the largest number of patients, written by Yan et al [36], reviewed the cases of 116 pregnant women and concluded that there is no evidence of increased risk of maternal death, spontaneous abortion, or preterm birth. Three single-center reviews with a small number of patients diagnosed with mild SARS-CoV-2 pneumonia (31 patients) came to the same conclusion, as none of them reported maternal deaths, intensive care unit admissions, nor poor perinatal outcomes [26,30,52]. In contrast, Zaigham et al [33] presented a systematic review of 108 patients, including 3 maternal intensive care unit admissions, 1 neonatal death, and 1 intrauterine death. Among these 20 most cited articles, the study by Hantoushzadeh et al [42] described the poorest maternal outcomes with 7 maternal deaths in a group of 9 pregnant women with severe SARS-CoV-2 pneumonia. It should be noted that most of the patients included in these publications were in the third trimester of pregnancy, and that no control groups were studied. In addition, some of the articles included cases of COVID-19 diagnosed by clinical criteria, without molecular (polymerase chain reaction) testing.

The imaging test used for diagnosis in the studies mentioned above was consistently chest CT. Two of the most cited articles presented in the second group identified after applying the clustering function addressed a radiation-free exam as a new alternative that could be particularly beneficial to pregnant patients. These publications were written by Moro et al [53] and Buonsenso et al [54], and propose using lung ultrasound examinations to recognize pathological patterns, some of which are especially suggestive of COVID-19 infection.

At the end of 2020, there was an upward trend in the numbers of studies that evaluated the effect of the COVID-19 pandemic on the mental health of women during pregnancy and the perinatal period. Studies conducted in Qatar [55], Iran [56], and the United States [57], and a meta-analysis by Hessami et al [58] provide evidence that the COVID-19 pandemic significantly increases the risk of anxiety among women during these periods. They concluded that these findings can be used to inform public health interventions, among which, consideration should be given to routine mental health screening of vulnerable groups and support measures for susceptible populations.

Regarding birth, some colleges of obstetricians such as the Royal College of Obstetricians and Gynaecologists in the United Kingdom initially developed guidance in March 2020 and affirmed that the delivery mode should be determined primarily by obstetric indication. They also recommended against routine separation of affected mothers and their babies [35]. In the most cited publication of this topic group, Della Gatta et al [46] stated that most pregnant patients with COVID-19 gave birth preterm by cesarean delivery, some of them on an elective basis. In most cases, the indication for cesarean delivery was not clearly specified, and it is certainly possible that the decision was influenced by the understandable anxiety toward the potential consequences of a new viral infection. It should be considered that this study was published in April 2020, when the available literature around the obstetric implications of COVID-19 was still very limited. Nevertheless, the fact that this elective intervention contributed to the favorable neonatal outcomes observed seemed unlikely even then. In this respect, on a review of the mode of delivery between December 2019 and April 2020, Debrabandere et al [59] also concluded that COVID-19 status alone became a common indication for cesarean delivery early in the pandemic, based on an attempt of obstetricians to serve their patients in the safest way possible given the climate of constantly evolving guidelines. As the literature expanded with no evidence for the intrauterine vertical transmission of COVID-19 from infected pregnant mothers to their fetuses [26,60], studies suggesting that vaginal delivery was associated with a low risk of intrapartum SARS-CoV-2 transmission to the newborn were also published [61]. A review article published in June 2020 concluded that neither vaginal delivery nor cesarean section conferred additional risks, and there was minimal risk of vertical transmission to the neonate from either mode of delivery [62]. In conclusion, as some clinical guidelines originally recommended, currently, the delivery mode should be decided based on contemplating the obstetric conditions, considering a cesarean delivery because of COVID-19 only if the mother has severe illness.

The precocity and efficacy of the vaccines developed against COVID-19 has been the most significant advance against the pandemic. Data from vaccinated pregnant people and small prospective cohort studies have not shown harmful effects, and have demonstrated a maternal immune response and transfer of maternal antibodies across the placenta and into breastmilk to confer passive immunity against SARS-CoV-2 in newborns after maternal vaccination with mRNA vaccines [63-65]. Based on these increasingly reassuring data regarding the safety and efficacy of COVID-19 vaccines during pregnancy, as well as data that pregnancy itself is associated with an increased risk of severe infection, currently, all pregnant women are recommended to undergo COVID-19 vaccination [66].

The development of vaccines has not prevented the constant search for therapeutic medicines, both among existing drugs with different indications and in the development of new drugs. Several antiviral agents are being used and evaluated for the treatment of COVID-19. Remdesivir, molnupiravir, and a combination of PF-07321332 (nirmatelvir) and ritonavir (marketed under the name Paxlovid) are three antivirals with different mechanisms of action that have demonstrated efficacy in clinical trials in terms of different markers of disease progression [67]. Remdesivir has been used without reported fetal toxicity in some pregnant people with Ebola and Marburg virus disease [68], and is being used to treat pregnant patients with severe COVID-19 [69], although almost all randomized trials of the drug have excluded pregnant and breastfeeding people. To date, dexamethasone is the only proven and recommended experimental treatment for pregnant patients with COVID-19 who are mechanically ventilated or who require supplemental oxygen. Although hydroxychloroquine and lopinavir/ritonavir may be used during pregnancy and lactation within the context of clinical trials, data from nonpregnant populations have not shown a benefit [10]. In any case, for most of these drugs, studies on efficacy, safety, and tolerance in pregnant women are limited.

The number of citation network studies has been increasing, as this is a very accessible and intuitive method of analysis, which provides a global overview of the different fields of study within a specific topic. The COVID-19 pandemic context has led to an abundance of publications quickly since the beginning of the outbreak. From late 2020 through 2021, thousands of scientific papers have appeared on COVID-19. In early 2020, studies about the scientific literature on COVID-19 and bibliometric analyses were published to summarize the research hotspots and compile a review to provide a reference for researchers. From disease inception to March 1, 2020, the first corresponding authors of the publications were from 20 different countries and the papers were published in 80 different journals. Lou et al [14] performed a search in PubMed using the keyword “COVID-19,” and identified and analyzed the data, including title, corresponding author, language, publication time, publication type, and research focus. Their results showed that China provided a large number of research data for various research fields during the outbreak of COVID-19, and most of the findings played an important role in preventing and controlling the epidemic around the world, which is expected since the pandemic began in China, as mentioned above. A bibliometric analysis of publications in five high-impact journals indexed to the SCI-EXPANDED database was also published [15]. By June, *The Lancet* was the journal with the highest number of contributions, and China, the United States, and the United Kingdom were the most represented countries [15,16]. Consistently, these countries were also identified to have the highest number of publications on COVID-19 and pregnancy in our analysis. In a textual analysis of 5780 publications extracted from the Web of Science, Medline, and Scopus databases, the most common topics found were guidelines for emergency care and surgery, viral pathogenesis, and global responses in the COVID-19 pandemic [16]. In Italy, a systematic review and bibliometric analysis of the scientific literature on the early phase of COVID-19 was performed, but with limited international impact [17]. Most articles focused on the hospital and clinical management of COVID-19.

Furthermore, citation networks were used to investigate the strategic themes, thematic evolution structure, and trends of coronavirus during the first 8 months of COVID-19 in the Web of Science database in 2020 [18]. The thematic evolution structure showed that the themes were evolving over time. The results of the strategic diagram highlighted “chloroquine,” “anxiety,” “pregnancy,” and “acute respiratory syndrome,” among others, as the clusters with the highest number of associated citations. Citation network analysis has also been used on the subject of pediatrics, identifying publication trends and topic dissemination, and showing the relevance of publishing authors, institutions, and countries [19]. The studies were published in 969 different journals, headed by *The Lancet*, and the authors were from 114 different countries with the most productive countries being the United States, China, and Italy. Pediatric research about COVID-19 has mainly focused on the clinical features, public health issues, and psychological impact of the disease. This is one of the few publications that has performed a bibliometrics analysis on COVID-19 and a specific health care area. Most recently, the interdisciplinary status of coronavirus-related fields was investigated via CORD-19. To this end, bibliometric indicators of interdisciplinarity were calculated and a cooccurrence analysis method was applied [20]. The disciplinary diversity of COVID-19–related papers published from January to December 2020 showed an upward trend. This reflects that COVID-19 has had a major impact not only on health but also on economics, politics, and the environment. Therefore, coronavirus-related issues are more complicated and difficult to adequately address by relying on a single field.

Moreover, CitNetExplorer software allows for the analysis of all existing studies on a particular topic, enabling much more in-depth studies to be performed. This might change the way in which research is performed in the different fields of study.

### Conclusions

This study offers a specific and objective analysis of the main articles published on COVID-19 and SARS-CoV-2 during pregnancy. In addition, it was possible to visualize, analyze, and explore the most cited articles and citation networks existing to date using the Web of Science and CitNetExplorer databases.

In light of above, we can conclude that articles that make up the bibliographic reference for knowledge about COVID-19 and pregnancy at this point lack some qualities such as unification of diagnostic criteria, a high number of patients, and comparisons with control groups. Furthermore, the possibility of using specific alternatives for pregnancy, such as lung ultrasound for diagnosis, is not widely described.

Consequently, COVID-19 is a relevant field for researchers, with the number of publications continuously on the rise. Owing to the accumulation of scientific knowledge, we have been able to understand the clinical features during pregnancy and the effect on perinatal outcomes. Recent studies have also provided data regarding the safety and efficacy of COVID-19 vaccines during pregnancy. With respect to drugs for treatment of the disease, studies on efficacy, safety, and tolerance in pregnant women are limited. These results could form the basis for further research and guide decision-making in COVID-19 research and treatments. Future bibliometric and scientometric studies on COVID-19 should provide updated information to analyze other relevant indicators in this field.

## Appendix

Multimedia Appendix 1 Additional data.

## Abbreviations

CitNetExplorer: Citation Network Explorer
CORD-19: COVID-19 Open Research Dataset
CT: computed tomography
MERS: Middle Eastern respiratory syndrome
SARS: severe acute respiratory syndrome
SCI-EXPANDED: Web of Science Core Collection’s Science Citation Index Expanded database
