# Supplementary material for: COVID-19 and Pregnancy: Citation Network Analysis and Evidence Synthesis
Source: JMIR Pediatr Parent. 2022 Mar 3;5(1):e29189. doi: 10.2196/29189 (PMC8989383; doi:10.2196/29189)
Supplement: Multimedia Appendix 1 [file pediatrics_v5i1e29189_app1.docx]

**Multimedia Appendix 1**

Language and countries

As for the language of the publications, 95.6% were in English, 1 1.5% in Spanish and 1.3% in German. Therefore, as shown in Table 1, the countries with the greatest number of publications are the United States (27.8%), China (13.9%) and England (9.0%).

| **Country** | **Publications (%)** | **Centrality** | **Degree** | **HalfLife** |
| --- | --- | --- | --- | --- |
| United States | 346 (26.0%) | 0.04 | 16 | -0.5 |
| China | 175 (13.1%) | 0.05 | 12 | -0.5 |
| England | 107 (8.0%) | 0.27 | 41 | -0.5 |
| Italy | 105 (7.9%) | 0.02 | 12 | -0.5 |
| Spain | 69 (5.2%) | 0.05 | 14 | -0.5 |

**Table A1.** Top 5 countries with highest number of publications

Research areas

The research area is multidisciplinary. The area of obstetrics and gynecology (33.2%) and internal medicine (14.9%) should be highlighted (Table 2).

| **Category** | **Frequency** | **Centrality** | **Degree** | **HalfLife** |
| --- | --- | --- | --- | --- |
| Obstetrics and Gynecology | 442 | 0.24 | 22 | -0,5 |
| Internal Medicine | 199 | 0.14 | 13 | -0,5 |
| Pediatrics | 128 | 0.12 | 11 | -0,5 |
| Environmental and occupational public health | 105 | 0.18 | 19 | -0,5 |
| Reproductive Biology | 82 | 0.09 | 8 | -0,5 |
| Experimental Medicine | 81 | 0.06 | 11 | -0,5 |
| Infectious Diseases | 77 | 0.16 | 11 | -0,5 |
| Immunology | 56 | 0.07 | 10 | -0,5 |
| Radiology, Nuclear Medicine, and Medical Imaging | 37 | 0.02 | 5 | -0,5 |
| Endocrinology and metabolism | 31 | 0.09 | 8 | -0,5 |

**Table A2.** Top 10 research areas with highest number of publications

Authors

As shown in table 3, the authors with the highest number of publications on COVID-19 and pregnancy are Baud D (0.95%), Birol P (0.82%) and Picone O (0.82%).

| **Author** | **Number of publications** | ***H***  **Index** | **Total**  **cites** | **Average of cites** | **Centrality** | **Degree** |
| --- | --- | --- | --- | --- | --- | --- |
| Gyamfi-bannerman C | 12 | 9 | 514 | 42.83 | 0.00 | 19 |
| Chen L | 11 | 7 | 1044 | 95.27 | 0.00 | 5 |
| Feng L | 11 | 4 | 277 | 39.57 | 0.02 | 9 |
| Goffman D | 11 | 7 | 448 | 40.73 | 0.00 | 11 |
| Baud D | 10 | 7 | 399 | 39.90 | 0.00 | 8 |
| Picone O | 10 | 7 | 146 | 16.30 | 0.01 | 8 |
| Jamiesson DJ | 9 | 6 | 506 | 60.00 | 0.04 | 9 |
| Sun GQ | 9 | 6 | 525 | 70.22 | 0.04 | 9 |
| Deruelle P | 8 | 6 | 161 | 23.25 | 0.00 | 4 |
| Ferrazi E | 8 | 6 | 216 | 30.63 | 0.00 | 4 |

**Table A3.** Top 10 authors with highest number of publications

Institutions

The institutions with the greatest number of publications, table 4, are Huazhong University of Science and Technology (5.14%), Wuhan University (3.13%) and University of Toronto (2.04%).

| **Category** | **Frequency** | **Degree** | **Centrality** | **HalfLife** |
| --- | --- | --- | --- | --- |
| Huazhong University of Science and Technology | 47 | 10 | 0.02 | -0,5 |
| Wuhan University | 26 | 10 | 0.03 | -0,5 |
| University of Toronto | 22 | 7 | 0.02 | -0,5 |
| University of Milan | 21 | 10 | 0.27 | -0,5 |
| Pekin University | 16 | 11 | 0.04 | -0,5 |
| Shanhai Jiao Tong University | 16 | 15 | 0.04 | -0,5 |
| University of Sao Paulo | 15 | 8 | 0.02 | -0,5 |
| Monash University | 15 | 12 | 0.06 | -0,5 |
| University of Paris | 14 | 9 | 0.16 | -0,5 |
| Kings College London | 14 | 7 | 0.00 | -0,5 |

**Table A4.** Top 10 Institutions with highest number of publications

Journals

Table 5 shows the main journals that have published on COVID-19 and pregnancy and the number of publications according to the WoS database.

| **Journal** | **Total publications** | **Impact Factor**  **(2019)** | **Quartile Score** | **SJR (2019)** | **Cites /Docs**  **(2 years)** | **Total Cites**  **(2019)** | **Centrality** | ***H***  **Index** | **Country** |
| --- | --- | --- | --- | --- | --- | --- | --- | --- | --- |
| *International Journal of Gynecology and Obstetrics* | 50 | 2.21 | Q2 | 0.83 | 1.995 | 1849 | 0..00 | 93 | United Kingdom |
| *Trials* | 45 | 1.88 | Q3 | 0.98 | 2.063 | 4135 | 0.00 | 72 | United Kingdom |
| *Journal of maternal fetal neonatal medicine* | 39 | 1.74 | Q3 | 0.70 | 1.812 | 3282 | 0.00 | 75 | United Kingdom |
| *American journal of perinatology* | 31 | 1.45 | Q3 | 0.79 | 1.485 | 1096 | 0.00 | 63 | United States |
| *European journal of obstetrics gynecology and reproductive biology* | 27 | 0.87 | Q3 | 0.80 | 2.145 | 2603 | 0.00 | 95 | Ireland |
| *Ultrasound in obstetrics gynecology* | 19 | 5.57 | Q1 | 2.85 | 6.049 | 3455 | 0.00 | 134 | United States |
| *American Journal of reproductive immunology* | 15 | 1.45 | Q3 | 1.22 | 3.035 | 1374 | 0.00 | 91 | United Kingdom |
| *Bjog an international journal of obstetrics and gynaecology* | 15 | 4.66 | Q1 | 2.00 | 4.398 | 3193 | 0.00 | 156 | United Kingdom |
| *American Journal of obstetrics and gynecology* | 12 | 6.50 | Q1 | 3.25 | 6.479 | 7320 | 0.00 | 216 | United States |
| *Journal of medical virology* | 12 | 2.02 | Q4 | 0.85 | 2.104 | 1758 | 0.00 | 111 | United States |

**Table A5.** Top 10 journals with highest number of publications

Keywords

On the other hand, the most used keywords have been "COVID-19" (390 publications), "Pregnancy" (281 publications) and "SARS-CoV-2" (156 publications). Table 6 and Figure 1, show the most used keywords in the most relevant publications.

| **Keyword** | **Frequency** | **Centrality** | **Degree** | **Total link strength** |
| --- | --- | --- | --- | --- |
| Covid-19 | 390 | 0.09 | 16 | 1454 |
| Pregnancy | 281 | 0.01 | 9 | 1168 |
| SARS-CoV-2 | 156 | 0.00 | 5 | 702 |
| Coronavirus | 128 | 0.00 | 7 | 587 |
| Women | 72 | 0.03 | 7 | 383 |
| Pneumonia | 756 | 0.04 | 14 | 375 |
| Infection | 56 | 0.03 | 7 | 228 |
| Vertical transmission | 44 | 0.04 | 8 | 262 |
| Protocol | 42 | 0.01 | 5 | 118 |
| Sars | 41 | 0.02 | 10 | 275 |
| Pandemic | 41 | 0.01 | 4 | 214 |
| Randomised controlled trial | 32 | 0.04 | 7 | 94 |
| Newborn | 32 | 0.02 | 6 | 150 |
| Mother | 32 | 0.02 | 5 | 212 |
| Pregnant women | 31 | 0.00 | 2 | 99 |
| Neonate | 29 | 0.00 | 4 | 107 |
| Acute respiratory syndrome | 29 | 0.03 | 12 | 125 |
| Covid 19 | 26 | 0.00 | 0 | 15 |
| China | 24 | 0.00 | 5 | 152 |
| Risk | 23 | 0.00 | 2 | 98 |
| Anxiety | 22 | 0.01 | 6 | 89 |
| Management | 21 | 0.00 | 3 | 101 |
| Outbreak | 19 | 0.00 | 5 | 120 |
| Coronavirus disease 2019 | 18 | 0.00 | 1 | 99 |
| Breastfeeding | 18 | 0.02 | 6 | 79 |
| Wuhan | 17 | 0.01 | 7 | 111 |
| Virus | 17 | 0.00 | 7 | 109 |
| Transmission | 17 | 0.01 | 6 | 123 |
| Depression | 17 | 0.00 | 4 | 68 |
| Influenza | 16 | 0.03 | 6 | 66 |

**Table A6.** The 30 most used keywords


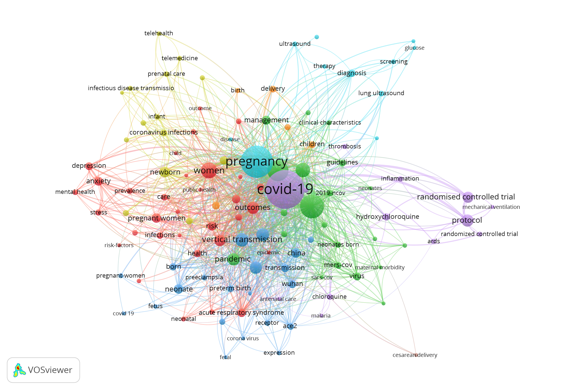


**Figure A1.** Co-occurrence between keywords

Table A7 shows the main characteristics of the 5 most important groups in Figure A1.

**Table A7.** Characteristics of the most used keywords

| **Cluster** | **Color** | **Main Keywords** | **Topic** | **%** |
| --- | --- | --- | --- | --- |
| 1 | Red | Women, outcomes, pregnant women, anxiety, depression | Psychological effects | 27.11 |
| 2 | Green | Sars-cov-2, coronavirus, pneumonia, management, virus | Clinical features | 17.80 |
| 3 | Blue | Vertical transmission, infection, china, sars, neonate | Vertical transmission of COVID-19 in late pregnancy | 17.80 |
| 4 | Yellow | Newborn, coronavirus infections, pandemics, obstetrics, infant | Obstetric and neonatal outcomes | 11.86 |
| 5 | Purple | Covid-19, protocol, randomized controlled trial, hydroxychloroquine, mechanical ventilation | Evaluation and treatment | 11.02 |
